# Maintenance Hemodialysis among Patients Visiting Nephrology Unit in a Tertiary Care Centre: A Descriptive Cross-sectional Study

**DOI:** 10.31729/jnma.7899

**Published:** 2022-11-30

**Authors:** Krishna Kumar Agrawaal

**Affiliations:** 1 Department of Internal Medicine, Nephrology Unit, Universal College of Medical Sciences, Siddharthanagar, Bhairahawa, Nepal

**Keywords:** *end-stage renal disease*, *hemodialysis*, *Nepal*

## Abstract

**Introduction:**

Maintenance hemodialysis is the treatment of renal failure and chronic kidney disease. Though the government of Nepal is providing free hemodialysis there is no chronic kidney disease registry or data to look into the clinical profile of patients. The aim of this study is to find out the prevalence of maintenance hemodialysis among patients visiting the nephrology unit in a tertiary care centre.

**Methods:**

This was a descriptive cross-sectional study carried out from 1 October 2021 to 15 October 2022. Ethical approval was taken from the Institutional Review Committee (Reference number: UCMS/IRC/132/22). All patients receiving maintenance hemodialysis were enrolled in the study. Convenience sampling was done. Point estimate and 95% Confidence Interval were calculated.

**Results:**

Among 2190 patients visiting the nephrology unit, maintenance hemodialysis was prevalent in 100 (4.57%) (3.70-5.44, 95% Confidence Interval).

**Conclusions:**

The prevalence of maintenance hemodialysis was found to be similar to the other studies done in similar settings.

## INTRODUCTION

The definition of chronic kidney disease (CKD) is based on the presence of kidney damage (eGFR <60 ml/min) for three months or more.^1^ The burden of CKD is escalating and so is End Stage Renal disease (ESRD). As the ESRD population will grow so will the need for Renal Replacement Therapy (RRT). Advances in RRT are astounding and Hemodialysis (HD) is still the most used modality all over the world. Maintenance hemodialysis is the treatment of renal failure and chronic kidney disease.

Hemodialysis is becoming safer but still, mortality among patients is high as compared to non-dialysis patients.^2^ There is yet no CKD registry in our country through the Government of Nepal providing free maintenance hemodialysis (MHD) services to its citizens.^3^ To provide better services it is imperative to look into clinico-demographic profile of patients at our centre.

The aim of this study is to find out the prevalence of maintenance hemodialysis among patients visiting the nephrology unit in a tertiary care centre.

## METHODS

This descriptive cross-sectional study was conducted from 1 October 2021 to 15 October 2022 under the aegis of the Nephrology Unit, Department of Internal Medicine at Universal College of Medical Sciences (UCMS). Ethical approval was taken from Institutional Review Committee (Reference number: UCMS/IRC/132/22). The patients visiting the Nephrology Unit during the study period were enrolled in the study. Convenience sampling was done and the sample size wascalculatedusing theformula:


$$\begin{array}{ll} \text{n} & ={\text{Z}}^{2}\times \frac{\text{p}\times \text{q}}{{\text{e}}^{\text{2}}}\\ & =1.96^{2}\times \frac{0.50\times 0.50}{0.03^{2}}\\ & =1068 \end{array}$$

Where,

n= minimum required sample size
Z= 1.96 at 95% Confidence Interval (CI)
p= prevalence taken as 50% for maximum sample size calculation
q= 1-p
e= margin of error, 3%

The minimum sample size calculated was 1068. However, the final sample size taken was 2190. The available clinical, demographic and laboratory parameters were recorded as per the proforma.

Data were analysed using IBM SPSS Statistics 17.0. Point estimate and 95% CI were calculated.

## RESULTS

Among 2190 patients visiting the nephrology unit, maintenance hemodialysis was prevalent in 100 (4.57%) (3.70-5.44, 95% CI). Among study subjects 54 (54%) were males and 46 (46%) were females with a male: female ratio of 1.17:1. In the study, 79 (79%), 11 (11%), 8 (8%) and 2 (2%) followed Hindu, Muslim, Buddhist and Christian religion respectively (Table 1).

**Table 1:** Sociodemographic profile (n= 100).

Characteristics	Categories	n (%)
Age group (years)	18-39	37 (37)
	40-59	39 (39)
	≥60	24 (24)
Sex	Male	54 (54)
	Female	46 (46)
Education status	Illiterate	37 (37)
	Literate without formal education	28 (28)
	Formal education	17 (17)
	High School (SLC)	14 (14)
	Intermediate (+2)	1 (1)
	Degree and above	3 (3)
Modified Kuppuswamy scale^4^	Upper lower	42 (42)
	Lower middle	37 (37)
	Upper middle	21 (21)
Religion	Hindu	79 (79)
	Muslim	11 (11)
	Buddhist	8 (8)
	Christian	2 (2)
Occupation	Farmer	24 (24)
	Home-maker	29 (29)
	Migrant worker	11 (11)
	Labourer	5 (5)
	Teacher	5 (5)
	Businessman	10 (10)
	Driver	6 (6)
	Wageworker	10 (10)
Body Mass Index (BMI) in kg/m^2^	<18.5	31 (31)
	18.5-23	45 (45)
	>23	24 (24)

**Figure 1:**
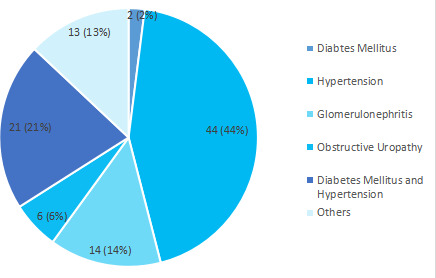
Comorbidities observed in patients on maintenance hemodialysis (n= 100).

The commonest cause of end-stage renal disease (ESRD) was hypertension 44 (44%) followed by diabetes mellitus 23 (23%). Among other comorbidities, tuberculosis was seen in 11 (11%) of the patients (Figure 1).

The mean pre-dialysis systolic and diastolic blood pressure was 140.69±21.26 mmHg and 85.69±10.48 mmHg respectively. The majority of the subjects, 60 (60%) were receiving twice-a-week maintenance hemodialysis. The minimum duration since dialysis was 5 months and the maximum duration was 7 years on MHD. The maximum length of dialysis session provided was 4 hours and the average duration of dialysis sessions per week was 8.67±1.51 hours. All the patients were dialyzed using the Polysulfone dialyzer with a surface area of 1.30 m^2^. Nearly half of our patients 45 (45%) were not on erythropoietin (EPO) injections and were receiving blood transfusions for anaemia (Table 2).

**Table 2:** Laboratory parameters of study population (n= 100).

Characteristics	Minimum	Maximum	Mean±SD	Median (inter-quartile range)
Hemoglobin (gm/dl)	6.00	12.50	9.10±1.55	9.00 (8.00-10.00)
Serum Albumin (mg/dl)	2.00	4.50	3.45±0.53	3.50 (3.00-4.00)
Serum Iron (mcg/dl)	13.00	201.00	93.31±43.47	88.70 (58.00-120.00)
Percent Iron Saturation (%)	5.00	120.00	37.44±21.56	34.00 (21.00-49.20)
Serum Uric acid (mg/dl)	1.50	11.60	5.51±1.84	5.40 (4.30-6.90)
Serum Phosphorous (mg/dl)	2.00	10.40	5.61±1.68	5.80 (4.50-7.00)
Serum Calcium (mg/dl)	5.00	12.00	8.13 ± 1.16	8.00 (7.00-9.00)

## DISCUSSION

The prevalence of patients on MHD was 4.5% among patients attending Nephrology services at UCMS. The mean age of our study population was 46.6 years which was slightly lower as compared to a study from Chitwan, Nepal (Mean age 58 years) whereas it was similar to another study from Kathmandu (Mean age 42 years).^5^ The most common cause of ESRD in the current study was Hypertension (44%) followed by Diabetes Mellitus (23%) which was similar to a study done in Nepal.^6,7^ However, this was different in two studies conducted at Bir hospital, Kathmandu where Chronic GN and Diabetes were major causes of CKD.^8,9^ The mean haemoglobin level in our study was 9.10±1.55 gm/dl. A study among 863 patients with ESRD for anaemia found up to 90% of patients with haemoglobin less than 10 g/dl.^10^ Similarly, a longitudinal analysis involving >65,000 dialysis patients showed only approximately 38% had haemoglobin levels within the range of 11 to 12 g/dl.^11^ Mineral and bone disorders are common complications seen in ESRD patients. The mean Serum Calcium level was 8.13±1.16 mg/dl. Dietary patterns and medical therapy are measures to correct hypocalcemia. This is similar to a study done at Gandaki Medical College.^12^ Thus, the clinical, demographic and laboratory profile of our patients was similar in context to studies from Nepal. The patients on MHD were regular due to the government of Nepal providing free HD services.

The limitation of our study is that it is a small-scale study and looked into basic clinical profiles only. So, the results can not be generalised.

## CONCLUSIONS

Majority of patients on hemodialysis are at low normal of the desired haemoglobin. Hypertension and diabetes are major comorbidities. Patients have low serum calcium levels.
